# Regadenoson in the rehabilitation of marginal donor lungs on ex vivo lung perfusion: A blinded multicenter randomized controlled clinical trial

**DOI:** 10.1016/j.xjon.2025.09.017

**Published:** 2025-09-20

**Authors:** Emily Fleischmann, Mark Conaway, Joseph Rabin, Kaitlyn Vecere, Kaitlyn Masih, Yunge Zhao, Ashling Zhang, Mohammad Khan, Valeria Mas, Alexander Krupnick, Joel Linden, Toshihiro Okamoto, Kamal Ayyat, Kenneth McCurry, Christine Lau

**Affiliations:** a Department of Surgery, University of Maryland School of Medicine, Baltimore, Md; b Division of Translational Research and Applied Statistics, University of Virginia, Charlottesville, Va; c Department of Medicine, University of Virginia, Charlottesville, Va; d Department of Thoracic and Cardiovascular Surgery, Cleveland Clinic, Cleveland, Ohio

**Keywords:** EVLP, ischemic reperfusion injury, primary graft dysfunction, regadenoson

## Abstract

**Objective:**

We hypothesized that regadenoson, an adenosine A2A receptor agonist, will increase the use rate after ex vivo lung perfusion and reduce ischemic reperfusion injury.

**Methods:**

This randomized (2:1), multicenter, blinded, placebo-controlled trial (NCT04521569) treated donor lungs with a regadenoson infusion (1.44 μg/kg/h, n = 26) or placebo (n = 8) during ex vivo lung perfusion. Eligibility criteria were adapted from the NOVEL trial. The rate of use of donor lungs was the primary end point. Secondary end points were primary graft dysfunction scores and 30-day safety.

**Results:**

Comparing regadenoson with placebo, 10 of 26 (38%) versus 0 of 8 (0%) donor lungs had a PaO_2_/Fio_2_ less than 300 (*P =* .04), the use rates were not significantly different with 17 of 26 (65%) and 6 of 8 (75%) lungs accepted after study treatment protocol (95% CI, −35 to 26; *P =* .62), and there was no significant difference in the proportion of recipients with at least 1 adverse event (12/17 [71%] vs 6/7 [86%], 95% CI, −25 to 42, *P =* .45), serious adverse event (11/17 [65%] vs 4/7 [57%], 95% CI, −36 to 51, *P =* .73), major lung event (10/17 [59%] vs 4/7 [57%], 95% CI, −34 to 59, *P =* .94), or grade 3 primary graft dysfunction (5/17 [29%] vs 2/7 [29%], 95% CI, −43 to 28, *P =* .87). No adverse events were related to the study treatment.

**Conclusions:**

There was no significant increase in the use of marginal donor lungs or a decrease in ischemic reperfusion injury in lungs undergoing ex vivo lung perfusion with regadenoson compared with placebo. Regadenoson is a safe ex vivo lung perfusion adjunct. The use rate of the placebo group was greater than the expectation set in the NOVEL trial of 51%, making it difficult for the regadenoson group to have a significant increase in use.

**Figure:**
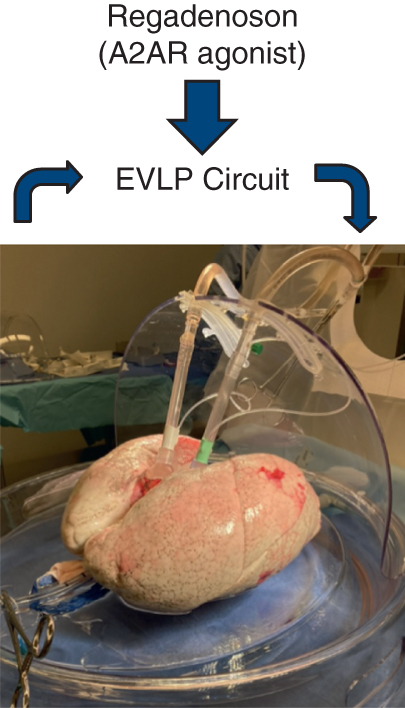
Regadenoson in the rehabilitation of marginal donor lungs undergoing EVLP.

**Central Message**

Regadenoson as an adjuvant to EVLP is shown to be safe in subsequent lung transplant recipients, although it does not increase the rate of use of marginal lungs at a dose of 1.44 μg/kg/h.

**Perspective**

Successful use of marginal donor lungs after EVLP can increase the availability of lung transplantation and presents an opportunity to combat IRI with adjunct therapies. Based on preclinical work, it is hypothesized that regadenoson, an A_2A_R agonist, will both increase use of marginal donor lungs undergoing EVLP and reduce IRI.

Lung transplantation is the preferred treatment for end-stage pulmonary disease; however, it has several limitations. The number of donor lungs has been steadily increasing, but demand continues to exceed the supply. The shortage of acceptable lungs leads to waitlist deaths and increased recipient mortality.^1^^,^^2^ The lung is the most susceptible to postmortem deterioration with only 18% of lungs used in multiorgan organ donors.^3^ Primary graft dysfunction (PGD) due to ischemic reperfusion injury (IRI) is associated with worse short- and long-term outcomes in lung transplant recipients. Any degree of PGD is correlated with an increase in the development of chronic allograft rejection.^4^^,^^5^

Ex vivo lung perfusion (EVLP) allows for the rehabilitation and functional assessment of donor lungs after procurement. This method has demonstrated the ability to increase the use of marginal lungs, which were previously discarded or not procured.^6^^,^^7^ Drugs, biologics, and other therapies are being investigated as adjuncts to EVLP to further increase use rates and lung quality.^8^

Adenosine acts on 4 receptors with the A_2A_ receptor (A_2A_R) having strong anti-inflammatory effects. A_2A_R agonists reduce immunologic response in the lungs via suppression of inflammatory cell activation and decreased cytokine release.^9^^,^^10^ A_2A_R agonists have been shown to decrease mean airway pressure, lung edema, and cytokines in porcine lungs undergoing EVLP.^11^ Additional studies of A_2A_R agonists in mouse and porcine models have demonstrated the attenuation of lung IRI.^12^^,^^13^ Regadenoson, a Food and Drug Administration–approved adenosine A_2A_R agonist, has been studied by our group and shown to be safe in the treatment of lung transplant recipients. In these patients, regadenoson decreases plasma levels of cytokines (interleukin-6, interleukin-8, and soluble form of the receptor for advanced glycation end products) and reduces PGD.^14^, ^15^, ^16^

On the basis of several preclinical and clinical studies, we hypothesized that regadenoson administered to donor lungs in conjunction with EVLP would increase the use rate of marginal donor lungs and reduce IRI in recipients. This has the potential to simultaneously address 2 limiting factors that have troubled the field of lung transplantation.

## Material and Methods

### Study Conduct

This multicenter, randomized, blinded trial was designed to assess the effect of regadenoson infusion on the rate of use of marginal lungs undergoing EVLP and the safety of subsequent lung transplantation in human recipients. The study was conducted at the University of Maryland School of Medicine (UMSOM) and Cleveland Clinic Foundation (CCF). Institutional Review Board (IRB) approval for the study protocol was obtained from the University of Maryland Baltimore IRB (HP-90075) on June 15, 2020, and Cleveland Clinic IRB (21-733) on August 20, 2021. All participants provided their written informed consent for publication before randomization. Randomization was performed by an unblinded study member via the University of Maryland Research Electronic Data Capture system. All study investigators and personnel delegated to lung evaluation and safety event review were blinded along with the participants. Donor lungs were randomized in a 2:1 ratio to EVLP with regadenoson or EVLP with placebo. Regadenoson (Lexiscan, Astellas Pharma US, Inc) was given at a dose of 1.44 μg/kg/h based on donor weight. The placebo was STEEN solution (XVIVO) given at a volume equivalent rate. The study dose was determined from preclinical data and clinical trials for lung transplant recipients and sickle cell anemia.^11^^,^^14^, ^15^, ^16^, ^17^ An investigational device exemption (G180190) for the use of regadenoson with EVLP was approved by the Food and Drug Administration. The study was overseen by a data safety monitoring board established by the National Heart Lung and Blood Institute. The study was registered at www.clinicaltrials.gov as NCT04521569 and conducted in accordance with the Declaration of Helsinki.

### Participants

Patients on the lung transplant waitlist were approached and provided written consent for publication. Donor lung eligibility was assessed before and after EVLP using criteria adapted from the XVIVO Perfusion System device labeling and NOVEL trial.^6^ Lungs were required to have a PaO_2_/Fio_2_ ratio 300 mm Hg or less or at least 1 donor risk factor (multiple blood transfusions, pulmonary edema, DCD, or high-risk donor history). Lungs were excluded for significant pneumonia, aspiration, mechanical injury, active infection, or single-lung EVLP. To be transplanted after EVLP, lungs were required to have a delta PaO_2_ more than 350 mm Hg at 2 consecutive time periods or meet 3 of 4 alternate criteria, stable or improved pulmonary vascular resistance, and compliance or airway pressure, and to be suitable for transplant in the opinion of the surgical investigator. Recipients were aged 18 to 75 years and excluded if they had preoperative extracorporeal membranous oxygenation, Burkholderia cepacia, prior lung transplant, or uncontrolled concurrent illness.

### Study Process

Donor lungs were allocated and procured following standard procedures. Randomization followed lung allocation before EVLP. EVLP was completed at Lung Bioengineering for the UMSOM and an on-site facility at CCF. EVLP was conducted according to standard protocol with the addition of the study infusion. The assigned treatment was administered by blinded study personnel via a syringe pump connected to the EVLP perfusate circuit beginning within 10 minutes of the start of EVLP, continuing for a minimum of 3 hours and a maximum of 4 hours. For lungs enrolled at UMSOM, perfusate and bronchoalveolar lavage (BAL) samples were collected during EVLP. Functional assessment was completed using delta PO_2_ (left atrial PO_2_ – pulmonary artery PO_2_), compliance, pulmonary vascular resistance, airway pressures, and radiographs. IRI was assessed via PGD grading according to the 2016 International Society for Heart and Lung Transplantation PGD definition.^18^ Recipients of study lungs were followed for 30 days after transplant.

### Study End Points

The primary end point was the use rate of evaluable lung, which is defined as the proportion of sets of lungs eligible for transplantation after undergoing EVLP with the study treatment. The secondary end point of the study was the safety of the subsequent transplants into human recipients for 30 days post-transplant. This was assessed by reporting adverse events including major lung events (MLEs).

### Laboratory Evaluation

Perfusate and BAL regadenoson concentrations were quantified via liquid chromatography-mass spectrometry by the University of Maryland School of Pharmacy Mass Spectrometry Center. Cytokine evaluation is presented in the Appendix E1.

### Study Design

The goal of this pilot study was to provide preliminary evidence of the effectiveness and safety of EVLP with regadenoson in rehabilitating marginal lungs. The sample size of 39 was based on an 80% chance of meeting “go/no-go” criterion to pursue a larger study if the use rate of lungs treated with regadenoson exceeded that with the placebo by at least 7.5% assuming the use rate with regadenoson and placebo was 70% and 50%, respectively. The NOVEL trial had a use rate of 51% and was used as a historic cohort to establish these assumptions.^6^ Lungs were considered evaluable if they met all eligibility criteria and none of the following occurred: not placed on EVLP, unexpected malfunction of the machine or drug not available. Allowing for a 15% proportion of nonevaluable lungs, the target sample size was 46. The 2:1 randomization ratio balanced the goals of having a high probability of making a correct “go” decision when regadenoson is effective, as well as having enough regadenoson-treated lungs to provide preliminary evidence of safety. The random allocation sequence was generated by an unblinded statistician and was stratified by site and randomly permuted blocks of random blocks sizes of 3 or 6 for the purpose of ensuring a 2:1 ratio.

### Study Monitoring

Use rates and grade 3 PGD for lungs receiving EVLP with regadenoson were monitored intermittently using a sequential probability ratio test for a binomial test of proportions. The lower boundary was used to protect against lower than expected use rates. Grade 3 PGD was monitored if regadenoson falsely elevated performance on EVLP with subsequent failure during transplant. Boundaries were based on the NOVEL trial.^6^

### Statistical Analyses

For the primary outcome, the differences between treatment groups in the proportion of lungs acceptable for transplantation were tabulated for each site along with standard 2-sample CIs.^19^ The Newcombe stratified risk estimate was used to combine estimated differences across sites. Similar analyses were done for the secondary outcome of grade 3 PGD, MLE, and other adverse event proportions. Comparisons among continuous variables were made using the nonparametric Wilcoxon test, with 95% CI for the Hodges-Lehmann shift estimate.

## Results

### Enrollment

The study enrolled patients at UMSOM from June 2021 to April 2024 and CCF from January 2022 to June 2024 when enrollment numbers were met. Study initiation was delayed by the COVID pandemic, especially at CCF.

**Figure 1:**
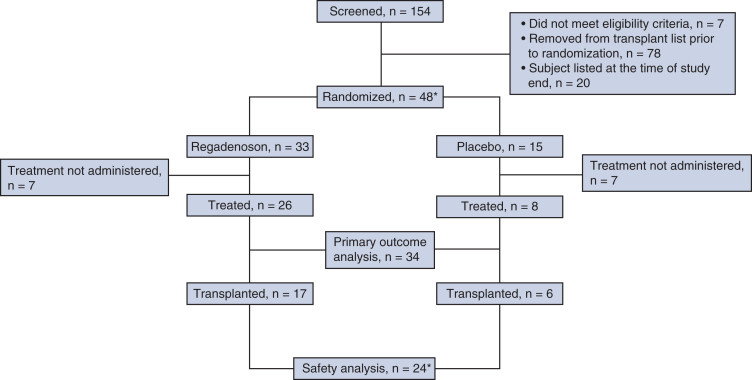
Participant scheme. ∗One pair of randomized lungs was split after EVLP, therefore accounting for 2 screening subjects and 2 safety analysis subjects.

The study screened 154 patients and randomized 48 pairs of lungs, of which 34 (regadenoson [n = 26] and placebo [n = 8]) underwent EVLP and were assessed for the primary outcome (Figure 1). A total of 23 pairs of lungs were accepted for transplant after EVLP. One pair of accepted lungs was split for 2 single lung recipients, therefore totaling 24 recipients for safety analysis. Of the subjects not randomized, 78 were removed from the transplant list including those who received a transplant not requiring EVLP. A total of 14 pairs of randomized lungs were nonevaluable due to not undergoing EVLP or unavailability of the study treatment.

### Participant Characteristics

Lung donor demographics in the regadenoson and placebo groups were average age (47 vs 41 years, *P =* .28), female sex (50% vs 88%, *P =* .09), cause of death (anoxia 38% vs 63%, stroke 35% vs 13%, trauma 27% vs 25%, *P =* .40) and indications for EVLP being PaO_2_/Fio_2_ <300 (38% vs 0%, *P =* .04), multiple blood transfusions (8% vs 13%, *P =* .68), pulmonary edema (27% vs 13%, *P =* .40), donation after cardiac death (DCD) (54% vs 63%, *P =* .67), and high-risk donor history (12% vs 13%, *P =* .94) (Table 1). The cold ischemic time averaged 369 versus 389 minutes (*P =* .65), and the total time on EVLP was 196 minutes and 207 minutes (*P =* .43) for the regadenoson and placebo groups, respectively. Except for the P/F ratio indication (*P =* .04), there were no statistically significant differences between the donor groups.

**Table 1:** Donor demographics

Category	Regadenoson (n = 26)	Placebo (n = 8)	*P* value
Age, y, mean (SD)	47.0 (15.1)	40.5 (13.1)	.28
Sex (female)	13 (50%)	7 (87.5%)	.09
Cause of death			.40
Anoxia	10 (38%)	5 (62.5%)	
Stroke	9 (35%)	1 (12.5%)	
Trauma	7 (27%)	2 (25%)	
EVLP indication			
PaO_2_/Fio_2_ <300	10 (38%)	0 (0%)	.04
Multiple blood transfusions	2 (8%)	1 (12.5%)	.68
Pulmonary edema	7 (27%)	1 (12.5%)	.40
DCD	14 (54%)	5 (63%)	.67
High-risk donor history	3 (12%)	1 (12.5%)	.94
Cold ischemic time 1∗ Mean (SD)	369 (114)	389 (105)	.50
Duration of EVLP Mean (SD)	196 (43)	207 (29.8)	.43

*DCD,* Donation after cardiac death; *EVLP,* ex vivo lung perfusion.

∗ Time elapsed from donor crossclamp to EVLP start.

Demographics for lung recipients in the regadenoson and placebo groups were average age (60.8 vs 63.7 years, *P =* .54), female sex (59% vs 57%, *P =* .94), race (African American 12% vs 14%, White 88% vs 86%, *P =* .87), indications for transplant (chronic obstructive pulmonary disorder/emphysema 41% vs 43%, cystic fibrosis/bronchiectasis 0% vs 14%, interstitial lung disease/pulmonary fibrosis 47% vs 29%, and other 12% vs 14%, *P =* .26), Lung Allocation Score (LAS) (44.5 vs 33.9, *P =* .11), and composite allocation score (27.6 vs 33.1, *P =* .18).

### Lung Outcome Analysis

The rate of use of lungs undergoing EVLP with regadenoson versus placebo was 17 of 26 (65%) versus 6 of 8 (75%), respectively, which was not statistically significant (95% CI, −35 to 26), *P =* .62). Comparing the clinical outcomes of regadenoson and placebo, there was a significant difference in initial intubation duration (58.2 vs 34.5 hours, 95% CI, 3.6-72.5, *P =* .02), but no significant difference in the intensive care unit length of stay (13.5 vs 14.6 days, 95% CI, −10.1 to 24.5) *P =* .39) or total length of stay (55.6 vs 33.1 days, 95% CI, −9.8 to 44.1, *P =* .17) (Table 2).

**Table 2:** Recipient demographics and outcomes

Category	Regadenoson (n = 17)	Placebo (n = 6)	*P* value
Age, y	60.8 (8.0)	63.7 (4.5)	.54
Sex (female)	10 (59%)	4 (57%)	.94
Race			
African American	2 (12%)	1 (14%)	.87
White	15 (88%)	6 (86%)	
Indication for transplant			
COPD/emphysema	7 (41%)	3 (43%)	.26
CF/bronchiectasis	0 (0%)	1 (14%)	
ILD/pulmonary fibrosis	8 (47%)	2 (29%)	
Other	2 (12%)	1 (14%)	
LAS,∗ mean (SD)	46.4 (17.0)	33.5 (0.7)	.11
Composite allocation score,∗ mean (SD)	27.6 (6.5)	33.1 (3.0)	.18
Outcomes			
Duration of index intubation (h)			
Median (Q1-Q3)	58.2 (48.5-102.5)	34.5 (30.0-43.2)	.02
ICU admission duration (d)			
Median (Q1-Q3)	13.5 (8.2-30.3)	14.6 (4.6-24.4)	.64
Total length of stay (d)			
Median (Q1-Q3)	55.6 (28.5-70.7)	33.1 (15.3-65.4)	.17

*CF,* Cystic fibrosis; *COPD,* chronic obstructive pulmonary disorder; *ICU,* intensive care unit; *ILD,* interstitial lung disease; *LAS,* Lung Allocation Score.

∗ LAS is reported for subjects randomized before March 9, 2023, and composite allocation score is reported from this date to the end of the study.

### Safety Analysis

When comparing the regadenoson and placebo groups, there was no significant difference in the proportion of adverse events or the number of recipients experiencing at least 1 adverse event (12/17 [71%] vs 6/7 [86%], 95% CI, −25 to 42, *P =* .45), serious adverse event (SAE) (11/17 [65%] vs 4/7 [57%], 95% CI, −36 to 51, *P* = .73), MLE (10/17 [59%] vs 4/7 [57%], 95% CI, −34 to 39, *P =* .94), or grade 3 PGD (5/17 [29%] vs 2/7 [29%], 95% CI, −43 to 28, *P =* .87) (Table 3). The most common SAEs were coagulopathy, bleeding, and acute kidney injury (Table 4). Coagulopathy in 2 recipients was due to concomitant pericardiectomy and congenital heart repair. Two bleeding SAEs were due to oropharyngeal bleeding and intraoperative adhesions. The change in coagulation studies including partial thromboplastin time, prothrombin time, international normalized ratio, and platelets from baseline to after treatment across all subjects was not significant. No adverse events were deemed related to the study treatment. There were no patient deaths. Stopping boundaries for lungs accepted for transplant and frequency of grade 3 PGD were not met at any point in the study.

**Table 3:** Total adverse events

Type of adverse event	Regadenoson n = 17	Proportion per regadenoson recipient	Placebo n = 7	Proportion per placebo recipient
Total adverse events	46	2.71	13	1.86
SAE	32	1.88	9	1.29
MLE	17	1.00	5	0.71
Acute rejection	0	0.00	0	0.00
Bronchial complication	0	0.00	0	0.00
Major pulmonary related infection	0	0.00	0	0.00
Retransplant	0	0.00	0	0.00
PGD 3	5	0.29	2	0.29
Reintubation	3	0.18	1	0.14
Tracheostomy	7	0.41	2	0.29
Acute kidney injury	2	0.12	2	0.29
Hypotension	0	0.00	1	0.14
Tracheostomy or redo-trach	1	0.06	1	0.14
Infection	1	0.06	0	0.00
Leukocytosis	1	0.06	0	0.00
Enteral feeding	1	0.06	0	0.00
Bleeding	2	0.12	0	0.00
Coagulopathy	2	0.12	0	0.00
Neurologic	2	0.12	0	0.00
Hyperammonemia	1	0.06	0	0.00
Atrial fibrillation	1	0.06	0	0.00
Anemia	1	0.06	0	0.00
Nonserious adverse events	14	0.82	4	0.57
Infection	2	0.12	0	0.00
Seizure	1	0.06	0	0.00
Gastroparesis/ileus	3	0.18	1	0.14
Thrombocytopenia	1	0.06	0	0.00
Malnutrition	1	0.06	0	0.00
Leukocytosis	1	0.06	0	0.00
Skin ulceration/pressure injury	3	0.18	0	0.00
Fever	1	0.06	0	0.00
Coagulopathy	1	0.06	1	0.14
Deep vein thrombosis	0	0.00	1	0.14
Atrial fibrillation	0	0.00	1	0.14

*SAE,* Serious adverse event; *MLE,* major lung event; *PGD,* primary graft dysfunction.

**Table 4:** Incidences of subjects with at least 1 safety event

Event	Regadenoson (n = 17)	Placebo (n = 7)	95% CI for the difference	*P* value
Adverse event	12 (71%)	6 (86%)	−25 to 42	.45
SAE	11 (65%)	4 (57%)	−36 to 51	.73
MLE	10 (59%)	4 (57%)	−34 to 59	.94
Grade 3 PGD	5 (29%)	2 (29%)	−43 to 28	.87

*P* values from Cochran-Mantel-Haenszel test, adjusting for site. *SAE,* Serious adverse event; *MLE,* major lung event; *PGD,* primary graft dysfunction.

### Laboratory Analysis

**Figure 2:**
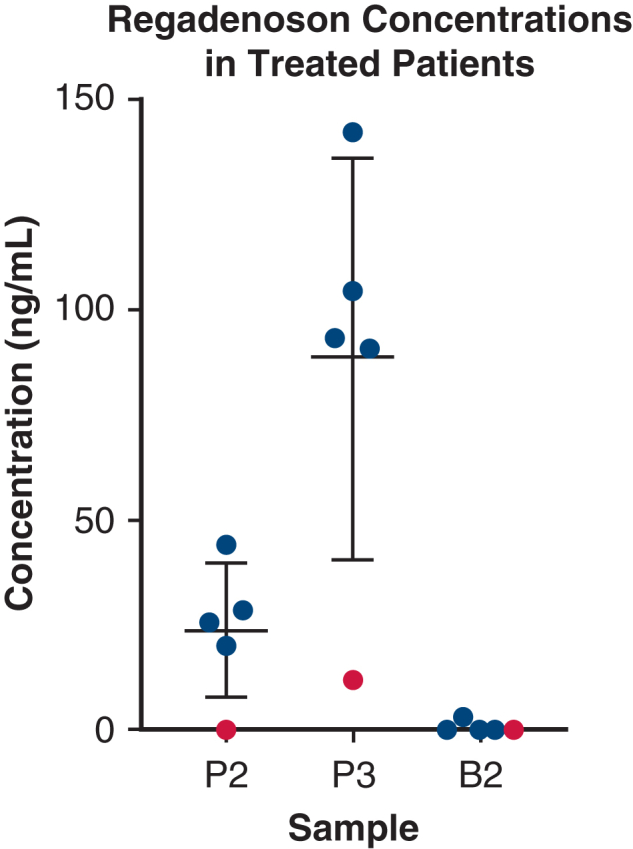
Regadenoson perfusate concentrations at 1 hour (P2), end of EVLP (P3), and BAL samples at the end of EVLP (B2) with scatter plot indicators for mean and SD of concentrations at each timepoint. One set of lungs in the treatment group exhibited very low regadenoson concentrations (0.00 ng/mL at P2, 12.19 ng/mL at P3), shown in red. This suggests a technical error during treatment infusion; therefore, regadenoson concentration data were excluded from analysis. One set of lungs was enrolled at UMSOM after regadenoson levels were analyzed and data are unavailable.

Regadenoson concentrations in the treatment group perfusate (n = 5) were 23.86 ng/mL (SD, 15.87) at 1 hour and 88.43 ng/mL (SD, 47.34) at the end of EVLP. The control group (n = 2) was consistently 0.00 ng/mL at all time points. Regadenoson was minimally detectable in BAL samples at the end of EVLP (0.82 ng/mL, SD, 1.52) (Figure 2). The results of cytokine sampling are shown in the Appendix E1.

## Discussion

This multicenter, randomized, blinded trial did not show a significant increase in the use of marginal donor lungs on EVLP receiving regadenoson compared with placebo; however, it demonstrated safety in the recipients.

The quality of lungs enrolled in this study covered a wide range in accordance with the adapted NOVEL trial criteria and thus added to the difficulty of comparing outcomes in a small pilot study. These generalizable criteria were chosen because of the need to include nearly all EVLP lungs with only 3% of transplants nationally and 12% at CCF undergoing EVLP.^7^^,^^20^ The NOVEL trial was a historical cohort with a use rate of 51%, but the regadenoson, placebo, and overall use rates of this trial were all far greater at 65%, 75%, and 68%, respectively. Additional comparison is made to the previously published use rate of 67% at CCF.^20^ The greater percentage of lungs in the placebo group versus the regadenoson group with a PaO_2_/Fio_2_ less than 300 (38% vs 0%, *P =* .02) suggests potentially higher-quality donor lungs. This is congruent with the unexpectedly high placebo acceptance rate of 75%. Lungs with a PaO_2_/Fio_2_ greater than 300 alternatively met eligibility via subjective high-risk donor criteria. The subjectivity of lung quality is a described challenge of EVLP. Current research is targeting the identification of objective indications for EVLP along with how to leverage EVLP data, such as biomarkers, radiography, and bronchoscopy to predict transplant outcomes.^8^

The 14 nonevaluable lungs negated the site stratification and permuted blocks, which along with the randomization rate of 2:1 contributed to the placebo sample size of n = 8 compared with n = 26 for the regadenoson group. The lung quality imbalance in the study groups was likely due to the small placebo sample size amplifying the results of every lung, which are subjective by nature. A 1:1 ratio would increase the placebo sample size, although it would have decreased safety data for the treatment group. Studying only DCD lungs would narrow the spectrum of lung quality and allow for more equal comparison.

A conservative treatment dose of 1.44 μg/kg/h was chosen because of concerns for cardiac effects; however, regadenoson levels on the EVLP circuit were approximately 100 times that of the prior in vivo study without any safety concerns identified.^14^, ^15^, ^16^ The drug level is due to the small volume of distribution on the EVLP circuit and absence of metabolism. The lungs are flushed before repackaging; thus, little regadenoson residual remains and there is no pharmacologic impact on the recipient. This is supported by the safety profile of the study, which did not meet the stopping boundary for rates of use or grade 3 PGD. Given this and the absence of related adverse events, regadenoson is safe to use as an adjuvant to EVLP. The intubation duration for the regadenoson group was longer than for the control group; however, the regadenoson-treated recipients had a higher LAS score and lower-quality lungs as described earlier. These factors could explain the intubation time differences, although direct comparison is difficult and the remainder of the results support the safety profile.

Murine models have utility in quantifying the residual drug concentration and guiding future dosing. Giving a bolus at the start of EVLP would maximize the time of perfusion at the target concentration, whereas the ex vivo platform decreases the concern for dose-limiting toxicities. Likewise, the use of antibodies, gene therapy, and viral-mediated treatments is being studied on the EVLP platform given the absence of interference and reduced hepatotoxicity advantage that selective organ treatment provides.^8^

## Conclusions

Regadenoson as an adjuvant to EVLP is shown to be safe in subsequent lung transplant recipients, although it does not increase the rate of use of marginal lungs at a dose of 1.44 μg/kg/h.

### Webcast

You can watch a Webcast of this AATS meeting presentation by going to: https://www.aats.org/resources/regadenoson-in-the-rehabilitat-9491.

**Figure:**
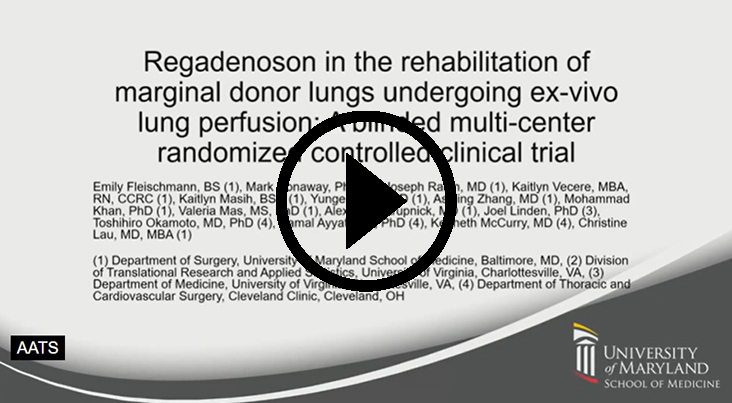

## Conflict of Interest Statement

The authors reported no conflicts of interest.

The *Journal* policy requires editors and reviewers to disclose conflicts of interest and to decline handling or reviewing manuscripts for which they may have a conflict of interest. The editors and reviewers of this article have no conflicts of interest.

## Figures and Tables

**Figure E1:**
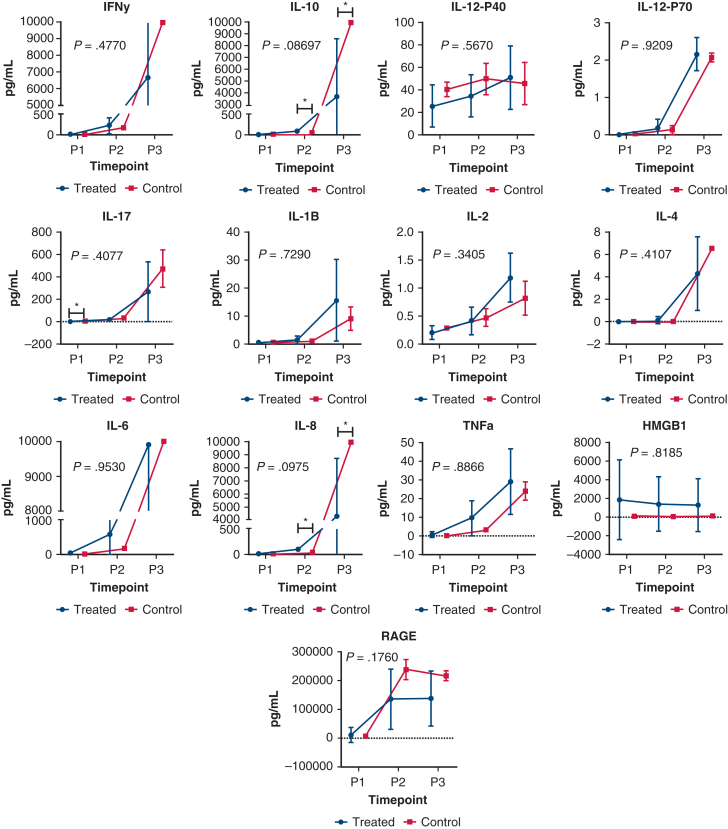
Concentrations of biomarkers detected in EVLP perfusate before treatment infusion (P1), 1 hour on EVLP (P2), and the end of EVLP (P3) in donor lungs on EVLP with regadenoson compared with EVLP alone. Each point represents the mean for all measurements taken at the specified time point within a treatment group. The symbols for the treatment and control groups at each timepoint are staggered along the x-axis. The *P* values listed are the interaction *P* values obtained from 2-way repeated-measures analysis of variance. Significant difference between treatment group at a specific timepoint are noted with an asterisk (∗).

## Appendix E1 Cytokine Analysis Methods

Cytokine and biomarker analyses of perfusate and BAL samples were performed by the University of Maryland School of Medicine's Cytokine Core Laboratory. The Luminex Multianalyte System (R&D Systems Inc) was used to quantify 11 cytokines/chemokines (interferon [IFN]-γ, interleukin [IL]-10, IL-12-p40, IL-12-p70, IL-17, IL-1b, IL-2, IL-4, IL-6, IL-8, and tumor necrosis factor [TNF]-α), and the biomarkers high mobility group box 1 and soluble receptor for advanced glycation end products were analyzed via a separate immunoassay. Standard repeated measures models were used to assess changes over time in cytokines levels in participants receiving lungs treated with regadenoson during EVLP.

### Cytokine Results

Biomarkers in perfusate and BAL samples were obtained for 7 treatment group lungs and 2 control group lungs. Nondetectable levels were represented numerically as 0.00 pg/mL, and levels greater than the maximum range were represented as the maximum detectable concentration (10,000 pg/mL, 2000 ng/mL, or 250,000 pg/mL). Biomarker levels were transformed to the log scale to stabilize variance. Across all biomarkers assessed (IFNγ, TNFα, IL-1b, IL-2, IL-4, IL-6, IL-8, IL-10, IL-12-p40, IL-12-p70, IL-17, high mobility group box 1, and soluble receptor for advanced glycation end products), there was no statistically significant relationship between treatment type and biomarker levels in the EVLP perfusate over time (Figure E1). Concentrations of the proinflammatory cytokines IFNγ, TNFα, IL-6, IL-8, and receptor for advanced glycation end products generally trended upward over time, which may be reflective of ongoing inflammation in the donor lungs related to IRI.
